# Supplementary material for: Nutritional status, symptom burden, and predictive validity of the Pt-Global web tool/PG-SGA in CKD patients: A hospital based cross sectional study
Source: PLOS Glob Public Health. 2023 Jan 17;3(1):e0001301. doi: 10.1371/journal.pgph.0001301 (PMC10022301; doi:10.1371/journal.pgph.0001301)
Supplement: Attachment — Submitted filename: Response to Reviewers.docx [file pgph.0001301.s002.docx]

**Dear Editor**,

We appreciate the time and thoughtful comments from the respected reviewers.

Please find the response to the reviewer comments as under;

**Reviewer #1**: Dear author(s),

Thank you very much for your high-quality paper on investigating the validity of the

nutritional assessment tools and the relevant outcomes for chronic kidney disease patients.

I have the following only two recommendations for your manuscript:

**1. line 87:** clarify the sentence - "...problems after nutritional." or "...problems after

nutritional assessment."

Response: It is problems after nutritional assessment. (corrected)

**2. Tables 1 and 4:** standardized to 1 d.p. for those zero results.

Response: All the Tables have been updated to 1 d.p. for those zero results.

**Reviewer #2:**

1. The study is not well organized; not understandable for the readers.

Response: We tried our best to revise the whole manuscript.

2. The objectives of the study are not well settled.

Response: Objective section was also modified.

3. The study has many outcomes/objectives as indicated indifferent parts/segments of it

Response: The study has three objectives/outcomes which includes nutritional status assessment, symptom burden (symptoms that mostly contribute malnutrition among CKD patients), and finally the predictive validity of Pt-Global web tool/PG-SGA.

4. The gap of the study is also not well dressed and not well focused.

Response: The evidence gap has been revised.

5. Study design is not clear/lack of consistency and appropriateness

Response: The study utilized cross-sectional approach. Methodology has been revised.

6. The analysis part need modification (when to use and report p- value, association report

writing and table association).

Response: Analysis part has been modified as advised.

7. The study finds also not clearly stated (not understandable)

Response: Study findings has been revised.

8. Since the above things are there, reviewing on discussion part is not done.

Response: Discussion part has also been revised.

9. Conclusion and Recommendation should be done on the study finds only in the precise form.

Response: Conclusion and recommendation part has also been modified.

10. Same of the references are not update.

Response: References updated.
