# Supplementary material for: Nutritional status, symptom burden, and predictive validity of the Pt-Global web tool/PG-SGA in CKD patients: A hospital based cross sectional study
Source: PLOS Glob Public Health. 2023 Jan 17;3(1):e0001301. doi: 10.1371/journal.pgph.0001301 (PMC10022301; doi:10.1371/journal.pgph.0001301)
Supplement: Attachment — Submitted filename: Response to Reviewers - R2.docx [file pgph.0001301.s003.docx]

**Dear Reviewer (#2)**,

We appreciate you once more for your valuable time and thoughtful remarks. Based on the feedback you provided during the initial revision, we completely modified the manuscript. While we made an effort to respond to a few of your comments again, other comments only make broad statements without identifying any specific comments. We would sincerely like to make the suggested corrections if there are any specific mistakes in the manuscript. Please find the response to the comments as under;

1. The study is not well organized; not understandable for the readers.

**Response:** We did our best to completely revise the manuscript (R1). Any further modification can change the dynamics of the research paper.

2. The objectives of the study are not well settled.

**Response:** Apologies, can you offer some guidance where is the problem. We sincerely hope to implement the suggested changes.

3. The study has many outcomes/objectives as indicated indifferent parts/segments of it

**Response:** The study has three objectives/outcomes which includes nutritional status assessment, symptom burden (symptoms that mostly contribute malnutrition among CKD patients), and finally the predictive validity of Pt-Global web tool/PG-SGA. Addressed in R1.

4. The gap of the study is also not well dressed and not well focused.

**Response**: All the three objectives are well explained in the manuscript.

**5.** Study design is not clear/lack of consistency and appropriateness

**Response**: The study utilized cross-sectional approach**. (Line number 102-221)**

**6.** Conclusion and Recommendation should be done on the study finds only in the precise

form.

**Response**: We have revised it again. (**Line number 421-428)**

**7.** Same of the references are not update

**Response: All the** references were thoroughly updated. **(Line number 452-636)**

8. Please edit your work (language)

**Response:** Edited. (Revision 1)
